# Asymmetric Drain Extension Dual-kk Trigate Underlap FinFET Based on RF/Analog Circuit

**DOI:** 10.3390/mi8110330

**Published:** 2017-11-09

**Authors:** Ke Han, Guohui Qiao, Zhongliang Deng, Yannan Zhang

**Affiliations:** 1 School of Electronic Engineering, Beijing University of Posts and Telecommunications, Haidian District, Beijing 100876, China; hanke@bupt.edu.cn (K.H.); dengzhl@bupt.edu.cn (Z.D.); murmures@bupt.edu.cn (Y.Z.); 2 School of Engineering, University of Edinburgh, Edinburgh EH9 3FF, UK

**Keywords:** underlap FinFET, asymmetric Dual-kk spacers, drain extension, short channel effects (SCEs), radio frequency (RF)/analog performance

## Abstract

Among multi-gate field effect transistor (FET) structures, FinFET has better short channel control and ease of manufacturability when compared to other conventional bulk devices. The radio frequency (RF) performance of FinFET is affected by gate-controlled parameters such as transconductance, output conductance, and total gate capacitance. In recent years, high-k spacer dielectric materials for manufacturing nanoscale devices are being widely explored because of their better electrostatic control and being less affected by short channel effects (SCEs). In this paper, we aim to explore the potential benefits of using different Dual-k spacers on source and drain, respectively: (AsymD-kk) trigate FinFET structure to improve the analog/RF figure of merit (FOM) for low-power operation at 14 nm gate length. It has been observed from the results that the AsymD-kk FinFET structure improves the coupling of the gate fringe field to the underlap region towards the source and drain side, improving the transconductance (*g_m_*) and output conductance (*g_ds_*) at the cost of an increase in Miller capacitance. Furthermore, to reduce the drain field influence on the channel region, we also studied the effect of asymmetric drain extension length on a Dual-kk FinFET structure. It can be observed that the new asymmetric drain extension structures significantly improve the cutoff frequency (*f_T_*) and maximum oscillation frequency (*f_max_*) given the significant reduction of inner fringe capacitance towards drain side due to the shifting of the drain extension’s doping concentration away from the gate edge. Therefore, the asymmetric drain extension Dual-kk trigate FinFET (AsymD-kk_DE_) is a new structure that combines different Dual-k spacers on the source and drain and asymmetric drain extension on a single silicon on insulator (SOI) platform to enhance the almost all analog/RF FOM. The proposed structure is verified by technology computer-aided design (TCAD) simulations with varying device physical parameters such as fin height, fin width, aspect ratio, spacer width, spacer material, etc. From comprehensive 3D device simulation, we have demonstrated that the proposed device is superior in performance to a conventional trigate FinFET and can be used to design low-power digital circuits.

## 1. Introduction

The introduction of FinFET technology has become an important milestone in the electronics industry. Commercially, Intel started using trigate FinFET technology at 14 nm [1], while most other semiconductor industries and foundries are expected to adopt FinFETs at 10/7 nm in the near future. Short channel effects (SCEs) are of serious concern in nano-scaled devices, affecting both radio frequency (RF) and analog performance [2,3,4]. Among the family of multigate structures, FinFET has the potential to suppress short channel effects, thereby enhancing the performance of RF/analog. Also, many of the advantages of FinFET technology come with several device-circuit co-design challenge [5]. Most of these challenges arise due to technological restrictions that degrade the short channel characteristics. While the introduction of underlaps improves the short-channel performance of the devices, drive current is reduced due to higher series resistance in the underlap regions. However, there is always a tradeoff between SCEs and source/drain (S/D) extension region resistance. A lot of work has been reported regarding the improvement on device SCEs of underlap FinFET with the help of S/D extension region engineering [6]. Moreover, high permittivity spacer materials have emerged as a potential performance booster to achieve better electrostatic control in ultra-scaled underlap devices [7].

At device level, several researchers have focused on the integration of high-k materials as a gate-dielectric or spacers [8,9,10,11,12,13,14]. Dual-k spacer double gate structure has been reported by [8] to control direct source to drain tunneling (DSDT) with improved SCEs. The fringe field phenomenon through these high-k gate dielectric has been studied by a few researchers from circuit perspectives in [9,10]. Pal et al. [11] have proposed that excellent control over channel and significant improvement in drive currents are achieved when employing Dual-k spacers in the underlap region. The use of Dual-k drain side spacers is proposed in [12] to increase leakage current *I_on_* without any significant degradation in outer fringe capacitance. A detailed capacitive analysis of symmetric and asymmetric Dual-k FinFETs for improved circuit delay metrics can be found in [13]. Singh et al. have highlighted a 14nm Analog and RF technology based on a logic FinFET platform for the first time and explored the direct impact of spacer engineering for RF/analog performance [14]. This paper presents a comprehensive study on 3D trigate FinFET to understand the effect of different asymmetric Dual-k spacers respectively and the asymmetric drain extension. Here, we propose that the use of different asymmetric Dual-k spacers and the asymmetric drain extension reflects the best improvement for higher drive current (on-off leakage current ratio *I_on_*/*I_off_*) and cutoff frequency (*f_T_*), with the disadvantages of parasitic capacitance increasing and slightly lower intrinsic gain (*A_v_*). A comprehensive study of optimal device parameters and RF/analog FOM due to permittivity spacers on circuit performances is still critically required.

This paper investigates the effect of using high-k spacers and its length from the circuit perspective and optimizes the device architecture for better RF/analog performance. Meanwhile, we have focused on variation of fin height (*H_fin_*), fin width (*W_fin_*), aspect ratio (AR = *H_fin_*/*W_fin_*) and Source/Drain extension length (*L_ext_*), studying its effect on RF/analog performance compared to conventional Dual-k spacer-based underlap SOI FinFETs. As these design parameters determine the FOM as well as other processing challenges, an analysis of the key parameters is crucial for achieving a reduction in device dimensions. The rest of the paper is arranged as follows. Section 2 briefly describes the asymmetric Dual-kk trigate FinFET device architecture and simulation methodology adopted. Device physics and RF/analog performance study aspects are designed and analyzed in Section 3. In Section 4, we propose a new structure—asymmetric drain extension (AsymD-kk)—and investigate its possible impact on the analog behavior of the device. Finally, Section 5 concludes the paper.

## 2. Asymmetric Dual-kk Trigate FinFET Structure and Performance Study 

**Figure 1:**
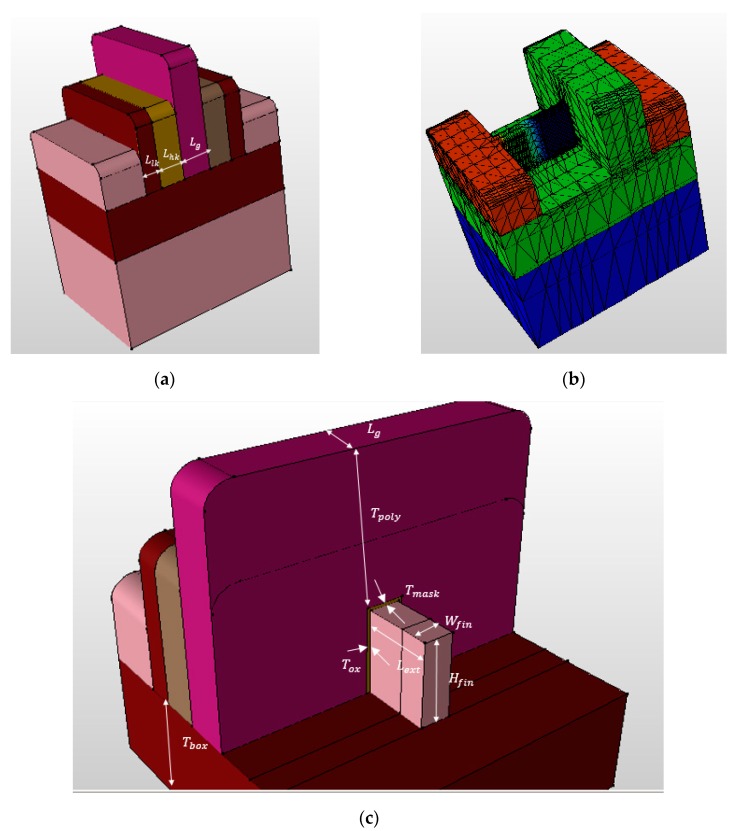
Schematic diagrams of AsymD-kk FinFE: (**a**) 3D view; (**b**) channel profile of FinFET source side; (**c**) cross section.

The asymmetric Dual-kk trigate underlap FinFET under study is shown in Figure 1. It consists of a different inner high-k on source (HfO_2_, 12 nm) and drain (Si_3_N_4_,12 nm), and outer low-k spacer material (SiO_2_, 8 nm) that contrasts with the different device structures, namely both side low-k spacers (conventional), both side Dual-k spacer (Dual-k), and source side only Dual-k spacer (Dual-kS). The physical and electrical parameters are calibrated to meet the specifications according to ITRS projections for 14 nm physical gate length (*L_g_*) [4]. Accordingly, the fin thickness (*W_fin_*), fin height (*H_fin_*), and equivalent oxide thickness (EOT) are 6 nm, 20 nm, and 0.8 nm, respectively. The metal-gate work functions are tuned to 4.45 eV for p-type to achieve a requisite threshold (*V_th_*) at a supply voltage of 0.9 V. Source/Drain (S/D) extension region uses Gaussian-doping profiles followed by a later doping gradient of 3 nm/decade. The S/D extension length (*L_ext_*) is taken as 20 nm (i.e., greater than the physical gate length). The channel and underlap regions are lightly doped with a boron concentration of 1 × 10^16^ cm^−3^ to reduce random dopant fluctuations (RDF) [13]. The raised source/drain regions have been formed to reduce the parasitic resistance associated with thin fins.

Moreover, to consider the gate-to-source/drain (G-S/D) capacitance, metal contacts are taken into consideration. The gate-electrode thickness (*T_g_*) is nearly twice the gate length value [7]. The inner high-k spacer (*L_hk_*) and outer low-k spacer length (*L_lk_*) are tuned to 12 nm and 8 nm, respectively, for an underlap length (*L_ext_*) of 8 nm. The thickness of the buried-oxide (BOX) layer is 25 nm. *T_mask_* represents the hard mask thickness on top of a silicon fin. *T_poly_* is the geometrical thickness of the gate material on top of the hard mask layer. *T_ox_* is the thickness of the gate oxidation. The nominal device parameters are listed in Table 1.

**Figure 2:**
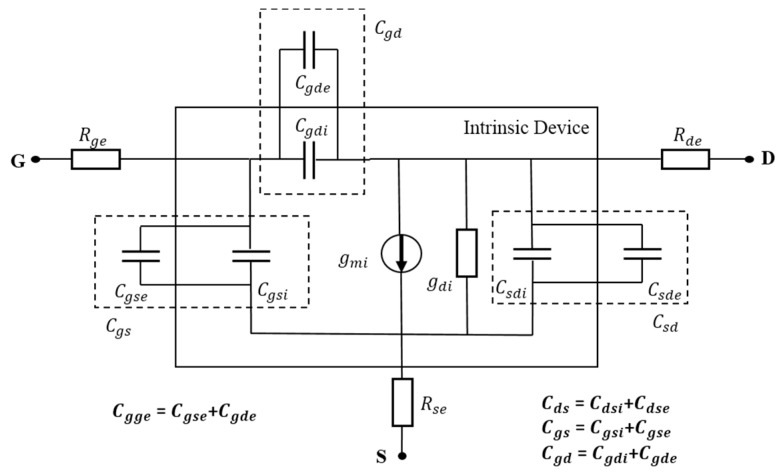
Small-signal equivalent circuit model.

Figure 2 shows the small-signal equivalent circuit model of FinFET, where the intrinsic elements *C_gsi_*, *C_gdi_*, *C_dsi_*, *g_di_*, and *g_mi_* are bias-dependent, whereas extrinsic capacitances *C_gse_*, *C_gde_* and *C_dse_* originate from the overlap between the source and drain regions and the thin gate oxide, and the fringing electric field between contacts. Also, the bias independent extrinsic series resistances *R_ge_*, *R_se_*, and *R_de_* are included. The intrinsic elements, which are parameters related to the physical phenomena inside the metal–oxide–semiconductor field-effect transistor (MOSFET) active region, depend on the geometry of the transistor and bias conditions [15]. The parasitic elements that surround the channel in order to get access to it are geometrically dependent but independent of the bias conditions. Recently, it has been established that the extrinsic gate capacitance is the main parameter responsible for the limited cut-off frequencies experimentally observed for triple-gate FinFETs [16].

Two RF figures of merit, namely, the current-gain cutoff frequency (*f_T_*) and the maximum oscillation frequency (*f_max_*), are evaluated using the following equation [17]:

(1) $$f_{T}=\frac{g_{mi}}{2\pi }\cdot \frac{1}{C_{gg\left(1+g_{di}R_{se}\right)}+C_{gde}R_{se}g_{mi}}$$

where *C_gg_* = *C_gge_* + *C_ggi_*, *g_mi_* and *g_di_* are the intrinsic transconductance and output conductance, respectively, and *R_se_* is the parasitic source resistance.

(2) $$f_{max}=\frac{\frac{g_{mi}}{2\pi C_{gs}}}{2\left(\frac{C_{gd}}{C_{gs}}\right)\sqrt{g_{di}(R_{se}+R_{ge})+\frac{1}{2}\frac{C_{gd}}{C_{gs}}\left(R_{se}g_{mi}+\frac{C_{gd}}{C_{gs}}\right)}}$$

The optimization of the fin geometry will have also an impact on *f_max_*, thanks to the reduction of the total extrinsic gate capacitance as well as the source and gate parasitic resistances. Therefore, optimal geometric parameters adjustments can produce improvements in *f_T_* and *f_max_*.

Three-dimensional simulations of devices were carried out using a TCAD 3D Sentaurus device simulator activating modified local-density approximation (MLDA) quantization model, a Lombardi mobility model accounting for mobility degradation at the semiconductor–insulator interface, a doping dependence SRH recombination or generation for deep defect levels at the gaps, a band to band auger recombination, and old slot boom bandgap narrowing phenomenon [18]. The RF/analog FOM are extracted at *I_ds_* = 10 μA/μm targeting weak/moderate inversion regime of operation. Cutoff frequency (*f_T_*) is extracted from current gain (h21) through an extrapolation of the –20 dB/decade slope, whereas maximum oscillation frequency (*f_max_*) is extracted from Mason’s unilateral gain (MUG) through an extrapolation of –20 dB/decade slope. The maximum oscillation frequency is a figure of merit related to the capability of the device to provide maximum available power gain at a large frequency [19]. Heavily doped raised source/drain regions are chosen for low parasitic resistance [20]. However, these do not affect the device performance significantly at such low drive currents. Gate height is chosen to be double *H_fin_* in accordance with the effective spacer formation step [21].

## 3. Design and Analysis of RF/Analogy Performance of AsymD-kk FinFET

The use of high-k sidewall spacers can better screen the gate fringe field towards the source/drain side over the underlap region. This increases the fringe field coupling between the gate and underlap regions and lowers the barrier of the underlap region in the strong inversion region [22]. For weak/moderate inversion, restricting the high-k spacer to underlap regions leads to a shifting in the lateral drain field at the gate edge towards the drain side, resulting in an improvement in *g_m_* and *g_ds_* [8]. Consequently, in this section various performance metrics like *I_on_*, *I_off_*, *g_m_*, *g_ds_*, *C_gg_*, *f_T_*, output resistance (*R_o_*), and gain (*A_v_*) are evaluated and the sensitivity of said parameters with *H_fin_*, *W_fin_*, and extended length (*L_ext_*) are systematically presented. Subsequently, we selected a fixed *L_ext_* of 20 nm with HfO_2_ and Si_3_N_4_ on source and drain, respectively, as inner high-k spacer length (*L_hk_*) and other varied parameters to study the effects of gate electrostatic integrity (EI) and in turn its effect on variations of RF/analog FOM of FinFET. Relying on the analysis of the experimental data, the AsymD-kk FinFET optimum parameters are selected and determined.

### 3.1. Fin Height (H_fin_)

Taller fins are required for high-drive current and matching the current drivability, whereas narrow fins ensure better SCE immunity. It is important to forecast improvement in *f_T_* and *f_max_* as traditional scaling of a FinFET is only achievable by choosing the optimal value of *H_fin_* and *W_fin_*. This phenomenon has been confirmed in [17,23]. However, manufacturing challenges and associated mechanical stresses are major concerns with taller fin devices. With increasing AR (*H_fin_*/*W_fin_*), the height may concentrate larger internal stresses in their relatively narrow base, causing fracture and in turn operational failure [24].

**Figure 3:**
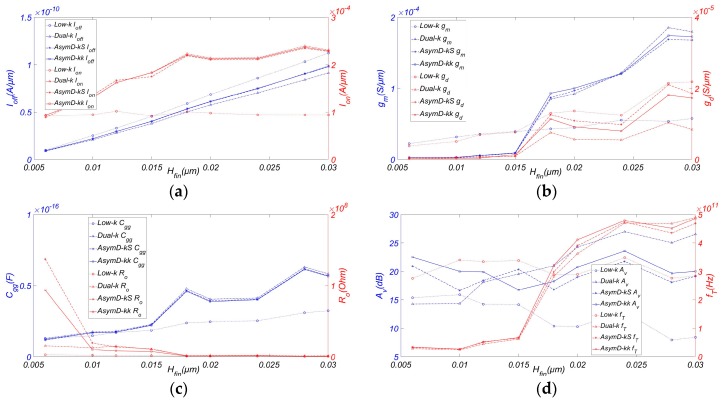
Variation of (**a**) *I_off_* and *I_on_* (**b**) *g_m_* and *g_ds_*, (**c**) *C_gg_* and *R_o_*, (**d**) *A_v_* and *f_T_* of FinFET with AR. Simulated with *W_fin_* = 6 nm, *L_ext_* = 20 nm, *T_ox_* = 0.8 nm, *L_hk_* = 12 nm, *σ_L_* = 3 nm, and *V_ds_* = 0.9 V.

Figure 3 plots the analog and RF FOM of AsymD-kk FinFET with varying AR compared to low-k, Dual-k, and Dual-kS FinFET structures. It is observed from Figure 3a that both *I_off_*, and *I_on_* are increasing with the increase in *H_fin_*. Hence, for higher current drivability and better SCE immunity, taller fins are required. When introducing the asymmetric Dual-kk spacer at source and drain side, the gate has more control over the channel, which results in a reduction in *I_off_* and improvement of *I_on_*. So it has been noticed that Dual-k structure shows a consistently higher *I_on_*/*I_off_* improvement (~1.6 times) in comparison with the conventional structure, followed by AsymD-kk and AsymD-kS structures. Moreover, AsymD-kk structure showed the highest improvement in *g_m_* by 50.39% with respect to the conventional structure, followed by AsymD-kS and Dual-k structures. Dual-k structure shows the lower value of *g_ds_* (~58.71%) in comparison to other structures. AsymD-kk and AsymD-kS structures show almost the same value of *g_ds_* at a height below 15 nm; subsequently, the AsymD-kk structure has a lower *g_ds_* and an improved *H_fin_*. 

Figure 3c shows the variation in *C_gg_* and output resistance *R_o_* with respect to fin height. The Dual-k structure shows the maximum *C_gg_* followed by AsymD-kS, AsymD-kk, and conventional structures. Moreover, AsymD-kS and AsymD-kk structures show almost the same value of *R_o_*, which is lower than that obtained for a Dual-k structure. This shows that the AsymD-kk structure has the potential to reduce parasitic feedback capacitance and thereby improves the gate control over the channel region, resulting in the reduction of SCEs at short channel lengths.

As shown in Figure 3d, the Dual-k structure shows the highest value of *A_v_*, followed by AsymD-kS, AsymD-kk, and conventional structures. However, the AsymD-kk structure shows a slight reduction (~14.49%) in *A_v_* in comparison with the Dual-k structure, but is higher than the AsymD-kS structure (about 8.48%). Compared to the increase in transconductance, the improvement of *g_ds_* is more obvious, resulting in a slight decrease. Meanwhile, as can be seen from the figure, the AsymD-kk structure has the best *f_T_* (~29.69%) in comparison with the conventional structure, followed by AsymD-kS and Dual-k structures. As we know, *f_max_* is inversely proportional to (*g_ds_* + 2π *f_T_C_gg_*)^1/2^ [25]; an increase in *f_T_* is counteracted by the reduction in *g_ds_* for the AsymD-kk structure in comparison to other structures. Therefore, *f_max_* is highest for the AsymD-kk structure with the variation of *H_fin_*. Considering the manufacturing challenges and limited performance improvements of taller fins, it is desirable to aim for a AsymD-kk structure FinFET with suitable fins (~20 nm) that outperforms the other structures in all FOMs.

### 3.2. Fin Width (W_fin_)

Due to the close proximity of multiple gates at smaller *W_fin_*, the longitudinal electric field at the source end of the device can be easily screened out, which increases the EI. However, as the transistors are scaled down, variations in critical transistor attributes such as *W_fin_* and *L_ext_* are becoming major issues in transistor design. The variations become larger as the feature sizes approach the fundamental dimensions such as the size of atoms and the wavelength of usable light for patterning lithography masks [26]. Of particular importance are RF/analog circuits, where device-level performance variation can make the specifications of the particular circuit fall below or rise above the desired value.

**Figure 4:**
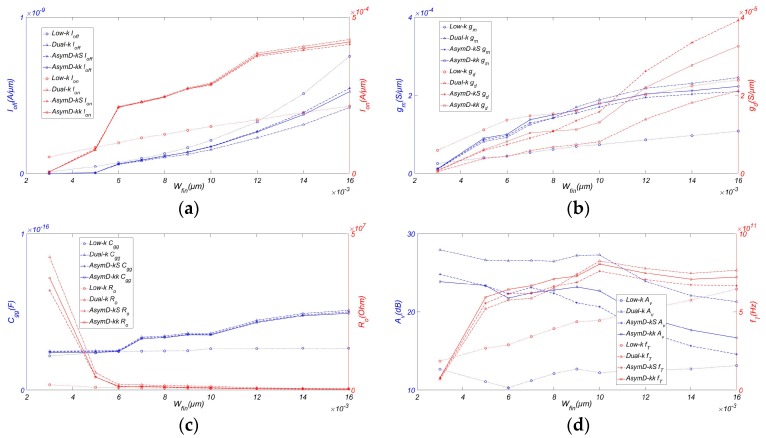
Variation of (**a**) *I_off_* and *I_on_*, (**b**) *g_m_* and *g_ds_*, (**c**) *C_gg_* and *R_o_*, (**d**) *A_v_* and *f_T_* of FinFET with AR. Simulated with *H_fin_* = 20 nm, *L_ext_* = 20 nm, *T_ox_* = 0.8 nm, *L_hk_* = 12 nm, *σ_L_* = 3 nm, and *V_ds_* = 0.9 V.

Figure 4 shows the variation of RF/analog FOM with *W_fin_*. At the aforementioned technology node, we have varied the *W_fin_* from 0.2 *L_g_* to 1.2 *L_g_*. By choosing a smaller *W_fin_*, we are able to minimize the longitudinal electric field. However, as scaling approaches the fundamental dimensions such as atomic size range and the sensitivity of the device, the parameters have a greater impact on the device performance, particularly in the case of RF/analog performance. It is observed that narrow fins are preferred for achieving higher *I_on_*/*I_off_* compared to low-k FinFET. From highest to lowest, we have Dual-k, AsymD-kk, and AsymD-kS structures. Also, we notice that *g_m_*, *g_ds_*, and *C_gg_* increase almost linearly with increasing *W_fin_*. The AsymD-kk structure has the highest improvement in *g_m_* (~51.38%) and the lowest value of *C_gg_* (~6.12%) in comparison to the conventional structure, followed by the AsymD-kS and Dual-k structures at a fin width of less than 10 nm. The Dual-k structure shows a lower value of *g_ds_* in comparison to other structures. However, the AsymD-kS and AsymD-kk structures show almost the same value of *g_ds_*. All designs show almost the same value of *R_o_* when varying *W_fin_*.

Figure 4d shows the variation in output resistance and *A_v_* with respect to the fin width. It can be noticed that the AsymD-kk structure has the highest improvement in *f_T_* (~25.11%) compared to the conventional structure, followed by the AsymD-kS and Dual-k structures. Meanwhile, despite the increase in *g_m_*, there is a large improvement in the *g_ds_* value for lower *W_fin_* values. The AsymD-kk structure shows a lower *A_v_* (~18.06%) compared to the Dual-k structure, but outperforms traditional structures 1.11-fold. In addition, the percentage improvement in *f_T_* of the AsymD-kk structure is limited below 0.5 *L_g_*. This may be attributed to the fact that the effective screening of gate fringing fields is improved with *W_fin_* scaling, thereby increasing the gate capacitance. Therefore, designing AsymD-kk FinFET with AR ~3 and *W_fin_* in the range 0.5–0.7 *L_g_* is a better option as compared to low-k FinFET.

### 3.3. Source/Drain Extension Length (L_ext_)

A similar type of analysis as that employed in the previous section was systematically discussed for the variation of *H_fin_* and *L_ext_*. An increase in underlap extension length (*L_ext_*) improved the gate controllability with reduced SCEs because of a shift in the lateral electric field from the gate edge toward the drain. Gate fringe-induced barrier lowering (GFIBL) has been observed in undoped underlap FinFET with an increase in the dielectric constant of the spacer region (*L_ext_*) [21]. The barrier to the lateral drain electric field is lowered in strong inversion because of an increase in the coupling of gate fringing fields to the undoped underlap portion of FinFET. As the spacer length is increased, more and more fringing fields are coupled to the underlap portion, thereby improving the short channel effects at low electron energies.

**Figure 5:**
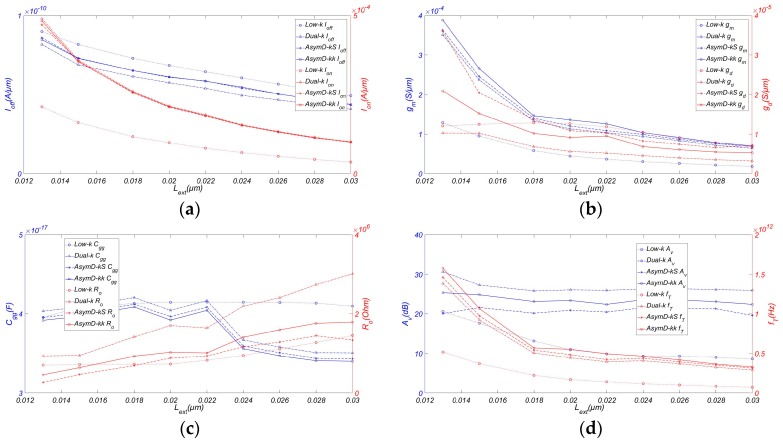
Variation of (**a**) *I_off_* and *I_on_* (**b**) *g_m_* and *g_ds_*, (**c**) *C_gg_* and *R_o_*, (**d**) *A_v_* and *f_T_* of FinFET with *L_ext_* Simulated with *H_fin_* = 15 nm, *W_fin_* = 6 nm, *T_ox_* = 0.8 nm, *L_hk_* = 12 nm, *σ_L_* = 3 nm, and *V_ds_* = 0.9 V.

Figure 5 plots the variation of RF/analog FOM with Source/Drain extension length (*L_ext_*). It is worth noting that the *I_off_*, *I_on_*, *g_m_* and *g_ds_* of both designs decrease linearly with an increase in *L_ext_* due to a reduction in gate control over the increased effective channel region. Compared to the conventional structure, the Dual-k structure is preferred to achieve higher *I_on_*/*I_off_*, followed by AsymD-kk and AsymD-kS, but the percentage improvement in *I_on_*/*I_off_* is reduced at higher *L_ext_* Additionally, AsymD-kk structures have the highest value of *g_m_* (~57.19%) and lowest value of *g_ds_* (~27.44%) with respect to the conventional structure. Simultaneously, AsymD-kk has a minimum value of *C_gg_* (~6.45%) and slightly larger *R_o_* compared to other structures. However, the total gate capacitance has a greater impact on the performance of the device than *R_o_*, and we can change other key device parameters to adjust the output resistance to the adaptive demand of the design.

It can be noticed from Figure 5d that the cut-off frequency *f_T_* of AsymD-kk FinFET is higher when varying *L_ext_* compared to its counterparts. From the chart, the percentage reduction in *C_gg_* is less than that found for *g_m_*, with an increase in *L_ext_* Therefore, AsymD-kk shows a slight reduction in *f_T_* with *L_ext_*, which is shown in Figure 5d. Meanwhile, the Dual-k structure shows the maximum gain *A_v_* compared to conventional structures, followed by AsymD-kS and AsymD-kk, but almost no variation with an increase in value of *L_ext_* Therefore, the FOM improvement of the AsymD-kk design is enhanced at approximately *L_ext_* ~20 nm, as shown in Figure 5.

### 3.4. Inner High-k Spacer Length (L_hk_) 

As the devices are scaled down to nano-scale regime formation, an ultra-shallow junction (USJ) can control the lateral electric field spread into the channel region [4]. The length of the inner high-k spacer has a direct impact on RF/analog FOM and formation of USJ. This is attributed to the fact that the fringing field screening via inner high-k spacer is more pronounced when the underlap portion near the gate edges remains undoped or lowly doped. To distinguish the effect of the high-permittivity spacer on underlap FinFET, *L_hk_* is varied from the gate to the source/drain edges for a fixed underlap length (*L_un_*) of 8 nm. Consequently, the same spacer extension length (*L_ext_* = *L_hk_* + *L_lk_*) of 20 nm is selected for AsymD-kk FinFET, selecting optimized *L_hk_* from 0 nm to 20 nm for analysis.

**Figure 6:**
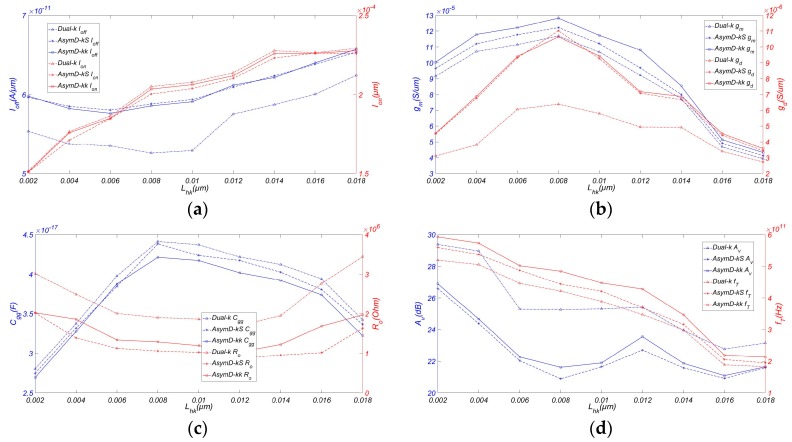
Variation of (**a**) *I_off_* and *I_on_*, (**b**) *g_m_* and *g_ds_*, (**c**) *C_gg_* and *R_o_*, (**d**) *A_v_* and *f_T_* of FinFET with *L_hk_*. Simulated with *H_fin_* = 15 nm, *W_fin_* = 6 nm, *T_ox_* = 0.8 nm, *L_ext_* = 20 nm, *σ_L_* = 3 nm, and *V_ds_* = 0.9 V.

It can be observed from Figure 6 that, on both designs, *I_off_* and *I_on_* increase linearly with increasing *L_hk_*. The parasitic resistance problem can be avoided by using higher *L_hk_*, which further increases the drain current and improves the SCE immunity. Meanwhile, by comparing the data in the figure, we can confirm that *L_hk_* ~12 nm represents the optimum predicted scenario. The AsymD-kk structure shows greater values of *g_m_* and *g_ds_* with respect to variation of *L_hk_* compared to the Dual-k and AsymD-kS structures. Furthermore, the AsymD-kk structure has a lower value of *C_gg_* and average *R_o_* compared with other structures. At this appropriate length (~12 nm), we can obtain the maximum *f_T_* and *A_v_*, and a relatively smaller gate capacitance. When compared to the Dual-k structure, the AsymD-kk structure has a lower *A_v_* (~7.35%) because the improvement in *g_m_* is found to be less than that of *g_ds_*. However, the AsymD-kk structure shows the highest value of *f_T_* (~18.79%). Also, the device acquires an appropriate threshold voltage (*V_th_*) and subthreshold slope (SS), which can better suppress the short channel effect and drain-induced barrier lowering (DIBL). Therefore, the FOM improvement of the AsymD-kk design is enhanced at approximately *L_hk_* ~12 nm, as shown in Figure 6.

## 4. Asymmetric Drain Extension Dual-kk Trigate Underlap FinFET (AsymD-kk_DE_)

From the previous analysis of AsymD-kk spacer structures, it was found that, with the exception of *A_v_*, all other analog parameters are better in the AsymD-kk structure than in the Dual-k structure. Simultaneously, it was found that the AsymD-kk structure outperforms the other two structures in terms of *g_ds_*, resulting in a slight reduction (~14.49%) of *A_v_*, despite having an increased value of *g_m_*. However, the AsymD-kk structure shows improvement in *g_m_*, *f_T_*, and *f_max_* and a reduction in *C_gg_* in comparison to the Dual-k and AsymD-kS structures. The main reason for the observed improvement of *g_ds_* compared with *g_m_* of the AsymD-kk structure is that there is less screening of the gate electric field towards the drain side since there is a lower high-k spacer towards the drain side in AsymD-kk structure, unlike in Dual-k structures. 

**Figure 7:**
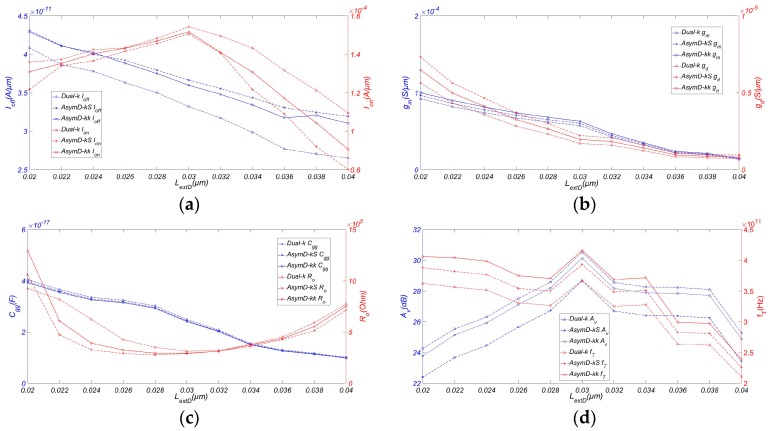
Variation of (**a**) *I_off_* and *I_on_*, (**b**) *g_m_* and *g_ds_*, (**c**) *C_gg_* and *R_o_*, (**d**) *A_v_* and *f_T_* of FinFET with *L_extD_*. Simulated with *H_fin_* = 15 nm, *W_fin_* = 6 nm, *T_ox_* = 0.8 nm, *L_ext_* = 20 nm, *σ_L_* = 3 nm, *L_hk_* = 12 nm, and *V_ds_* = 0.9 V.

To reduce the effect of the drain over the channel region and retain the Dual-k structure of the source and drain, we used an AsymD-kk_DE_ (asymmetric drain extension Dual-kk trigate underlap FinFET) structure. This section describes the potential benefits of using AsymD-kk_DE_ to further improving the analog/RF FOM. Figure 7a shows the variation of *I_off_* and *I_on_* with respect to the drain extension length (*L_extD_*) at a fixed source extension length (*L_extS_*) of 20 nm of the AsymD-kk_DE_ structure. As we increase *L_extD_* from 20 nm to 40 nm, a reduction in *I_off_* (~27.67%) and *I_on_* (~30.81%) can be observed. Moreover, AsymD-kk_DE_ shows a consistent *I_on_*/*I_off_* compared to the Dual-k structure. Additionally, it was shown to have a higher *I_on_*/*I_off_* improvement (~3.57%) in comparison with AsymD-kS. In addition, *g_m_* and *g_ds_* decrease from 10.131 μS/μm to 1.475 μS/μm (~85.25%) and 0.719 μS/μm to 0.084 μS/μm (~87.02%), respectively. 

The AsymD-kk_DE_ structure has lower values of *C_gg_* and *R_o_* compared to other structures when *L_extD_* has a value of approximately 30 nm. An increase in *L_extD_* shifts the drain doping away from the gate edge towards the drain side, resulting in a reduction of *C_gd_*. However, increasing *C_gg_* is due to fewer SCEs in the AsymD-kk_DE_ structure because of the increased *L_extD_* [27]. The combined effect of *C_gs_* and *C_gd_* translates into a decrease in *C_gg_* by 38.57%, with an increase in *L_extD_*, as shown in Figure 7d.

The reduction in *g_ds_* is more significant than for *g_m_* Therefore, Figure 7d shows a slight improvement in *A_v_* from 23.77 to 30.13 dB with an increase in *L_extD_* from 20 nm to 30 nm. This is followed by a slight reduction to 24.88 dB until the length of *L_extD_* reaches 40 nm. Moreover, the AsymD-kk_DE_ structure shows an improvement in *f_T_* (~2.48%) and *f_max_* with an increase in *L_extD_* from 20 nm to 30 nm. Subsequently, it goes slightly down as it approaches 40 nm. This shows that there is a range of values of *L_extD_* (~30 nm), which results in a better performance observed from simulating RF/analog devices.

To understand the contribution of asymmetric drain extension on the analog/RF performance of the AsymD-kk_DE_ structure, we also studied the Dual-k and AsymD-kS structures with asymmetric drain extension. As seen in the figure, the conventional structure with asymmetric drain extension has a better performance in almost all analog/RF FOM, except a slight reduction in *g_m_* and *g_ds_* in comparison to the conventional structure without drain extension. This occurs because of a significant reduction in inner fringe capacitance towards the drain side due to a shifting of the drain extension’s doping concentration away from the gate edge. It would not be appropriate to say that the improvement is produced by only an AsymD-kk spacer at the source or only by the asymmetric drain extension. However, observing the simulation results, the AsymD-kk structure shows more significant improvement in *g_m_* and *g_ds_*, and the asymmetric drain extension structure shows more significant improvement in *f_T_* and a lower gain reduction. If we compare the AsymD-kk structure to other structures, it shows superior values of *g_m_*, *g_ds_*, *f_T_*, and *f_max_*, with a slightly reduced value of *A_v_*.

## 5. Conclusions

Asymmetric drain extension Dual-kk trigate underlap FinFET is an attractive option for designing circuitry for 14 nm low-power and high-frequency battery-operated portable devices given the improved RF/analog FOMs that they offer. It has been found that an asymmetric Dual-kk spacer strongly affects the *g_m_* and *g_ds_* values of the device. Moreover, AsymD-kk structures show better performance in almost all analog/RF FOMs in comparison to the conventional structures for low-power operation with the exception of intrinsic gain, which was found to be lower. Therefore, further improvement in *A_v_* and *f_T_* of the AsymD-kk structure can be obtained by introducing asymmetricity in drain extension regions (the AsymD-kk_DE_ structure). From the simulations, it has also been observed that the AsymD-kk_DE_ structure shows an improvement in *g_m_* by ~9.09%, in *g_ds_* by ~13.04%, in *f_T_* by 12.91%, in *A_v_* by 19.47%, and also a reduction in *C_gg_* by 40.41% in comparison with the Dual-k structure at drain extension length *L_extD_* of 30 nm. Finally, from the reported results, it can be concluded that AsymD-kk_DE_ FinFET is outperformed as compared to Dual-k FinFET for designing 14 nm low-power and high-frequency RF/analog circuits or robust SRAMs in FinFET technology, just selecting the optimal structural parameters. In future work, we will study the effect of various K values on RF/analog circuits and the improvement of delay performance.

## Figures and Tables

**Table 1:** Nominal device parameters.

Parameters	Description	Typical Value/nm
*L_g_*	Gate length	14
*H_fin_*	Fin height	20
*W_fin_*	Fin width	6
*T_mask_*	Hard mask thickness over fins	0.8
*T_poly_*	Geometrical thickness of gate material over hard mask	15
*T_ox_*	Thickness of gate oxide	0.8
*L_ext_*	Source/Drain extension length	20
*L_hk_*	The inner high-k spacer length	12
*L_lk_*	outer low-k spacer length	8
